# Technical note: Cartilage imaging with sub‐cellular resolution using a laboratory‐based phase‐contrast x‐ray microscope

**DOI:** 10.1002/mp.16599

**Published:** 2023-07-11

**Authors:** Michela Esposito, Alberto Astolfo, Silvia Cipiccia, Charlotte Maughan Jones, Savvas Savvidis, Joseph D. Ferrara, Marco Endrizzi, Jayesh Dudhia, Alessandro Olivo

**Affiliations:** ^1^ Department of Medical Physics and Biomedical Engineering University College London London UK; ^2^ Diamond Light Source Harwell Science and Innovation Campus Didcot UK; ^3^ Rigaku Americas Corporation The Woodlands Texas USA; ^4^ The Royal Veterinary College Hatfield Hertfordshire UK

**Keywords:** cartilage imaging, cellular imaging, dark‐field, phase‐contrast imaging, soft tissue imaging, x‐ray microscopy

## Abstract

**Background:**

Microscopic imaging of cartilage is a key tool for the study and development of treatments for osteoarthritis. When cellular and sub‐cellular resolution is required, histology remains the gold standard approach, albeit limited by the lack of volumetric information as well as by processing artifacts. Cartilage imaging with the sub‐cellular resolution has only been demonstrated in the synchrotron environment.

**Purpose:**

To provide a proof‐of‐concept demonstration of the capability of a laboratory‐based x‐ray phase‐contrast microscope to resolve sub‐cellular features in a cartilage sample.

**Methods:**

This work is based on a laboratory‐based x‐ray microscope using intensity‐modulation masks. The structured nature of the beam, resulting from the mask apertures, allows the retrieval of three contrast channels, namely, transmission, refraction and dark‐field, with resolution depending only on the mask aperture width. An *ex vivo* equine cartilage sample was imaged with the x‐ray microscope and results were validated with synchrotron tomography and histology.

**Results:**

Individual chondrocytes, that is, cells responsible for cartilage formation, could be detected with the laboratory‐based microscope. The complementarity of the three retrieved contrast channels allowed the detection of sub‐cellular features in the chondrocytes.

**Conclusions:**

We provide the first proof‐of‐concept of imaging cartilage tissue with sub‐cellular resolution using a laboratory‐based x‐ray microscope.

## 1 INTRODUCTION

Imaging techniques play a pivotal role in both research and clinical practice for osteoarthritis (OA), including for diagnosis and monitoring of the disease, as well as studies in the etiology of the disease and the development of treatment. In vivo studies are typically performed with magnetic resonance imaging (MRI),^1^ as x‐ray techniques typically lack the contrast required to visualize cartilage. X‐ray radiology however remains the main approach in the clinic for OA which is visualized as articular joint space narrowing that typically manifests in advanced disease. At the microscopic scale however, which is particularly relevant for assessing re‐cellularization and growth in tissue engineered models, histology is the most commonly used technique. While histology can be considered the gold standard for many biological and medical applications, it lacks the volumetric information available in tomographic imaging and restricts the visualization to a fixed anatomical orientation. Additionally, as a destructive technique, it is affected by processing (embedding and cutting) artifacts in the plane of dissection and, thus, observation.^2^

Phase‐contrast x‐ray microscopy has the potential to overcome these limitations, by providing non‐destructive tomographic imaging with isotropic resolution in the micron length scale for samples in their native state. Phase‐contrast imaging relies on measuring the phase‐shift imparted by the sample to the x‐ray wave, as opposed to conventional x‐ray imaging which measures variations in x‐ray intensity (absorption contrast). Since changes in phase can be order of magnitude larger than changes in attenuation for soft tissue samples at relatively low x‐ray energies, an increased contrast for soft tissue is expected for phase‐contrast imaging compared to absorption imaging. Several studies have been published in the last two decades demonstrating the suitability of synchrotron‐based x‐ray phase‐contrast techniques for cartilage imaging, both in vivo^3^ and *ex vivo*.^4^, ^5^, ^6^, ^7^ Examples of laboratory‐based cartilage imaging at macroscopic level exist for several phase‐contrast imaging methodologies, including analyzer‐based imaging,^8^ grating interferometry,^9^ propagation‐based imaging^10^ and edge‐illumination.^11^ These studies mainly demonstrate the capability of phase‐contrast imaging to distinguish between cartilage and bone tissues, due to the increased contrast arising from measuring phase shifts. However, images reported in the aforementioned studies are unable to capture structural information within the cartilage. This is attributed to insufficient imaging system resolution as opposed to the underlying physical principles of these phase‐based imaging systems. More recently, Horng et al.^12^ reported on high‐resolution synchrotron‐based techniques for the visualization of cellular and sub‐cellular features in healthy and degenerated cartilage samples.

In this article, we report on the first proof‐of‐concept results of imaging *ex vivo* cartilage with sub‐cellular resolution using a laboratory‐based x‐ray phase‐contrast microscope. The laboratory results are validated with the gold standard synchrotron tomography, as well as histology. The complementarity of the retrieved contrast channels is also discussed with respect to cellular morphology.

## 2 MATERIALS AND METHODS

### 2.1 Sample preparation

A thin section (≈2×0.5×0.5 mm^3^) of equine articular cartilage was excised post‐mortem from the metacarpophalangeal joint. Horse tissues were obtained following approval from the Royal Veterinary College Clinical Research Ethical Review Board (URN 2020 2017‐2). The sample was fixed in 4% paraformaldehyde and dehydrated in 70% ethanol. The sample was transferred to a 1‐mm diameter glass capillary (Hampton Research) and kept in an ethanol atmosphere.

### 2.2 Synchrotron imaging

The cartilage sample was imaged at Diamond Light Source (DLS) on the I13‐2 beamline. A so‐called “pink” polychromatic beam with mean energy of 25 keV was used to perform tomographic imaging of the sample. A Pco.edge 5.5 camera with effective pixel size of 0.8 µm was used and placed at 20 cm from the sample, while the source to sample distance was 200 m. The scan, performed with continuous sample rotation in the so‐called fly‐scan mode, consisted of 1800 projection images over 180 degrees for a total scan time of 5 min. Data were reconstructed using a filtered‐back‐projection algorithm following application of Paganin's phase retrieval^13^ to all individual projections, implemented within the DLS Savu environment.^14^

### 2.3 Laboratory imaging

**FIGURE 1:**
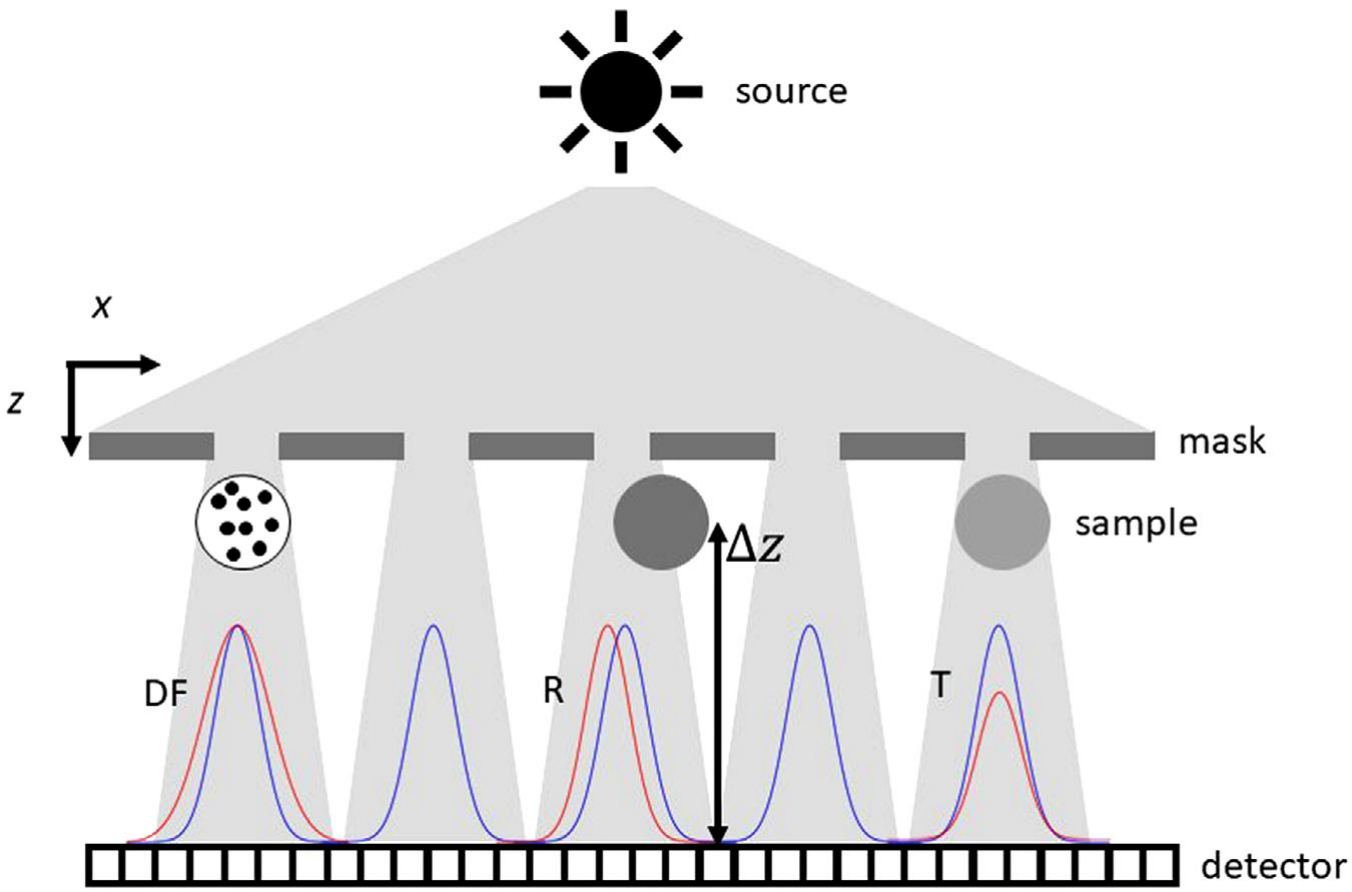
A schematic representation of the Beam Tracking (BT) principle. Profiles of shaped beamlets with (red) and without (blue) sample in place are shown, where changes in amplitude (T), position (R) and width (DF) of the beamlets are also highlighted.

Planar imaging of the cartilage sample was obtained with a laboratory‐based beam tracking (BT) system.^15^ The set‐up^16^ consists of a rotating Cu anode x‐ray source and a doubly‐curved multi‐layer monochromator, selecting the Cu K_α_ lines at 8 keV and focusing the beam into a 350‐µm focal spot. As schematically shown in Figure 1, the focused beam is structured into an array of beamlets by a 20‐ µm‐thick gold membrane featuring 2‐µm‐wide slits with a 20‐µm periodicity. The structured beamlets were imaged by a CMOS sensor, coupled to a scintillator and objective, with an effective pixel pitch of 1.1 µm and a field of view of 1.6× 1.6 mm^2^. A sample to detector distance of 15 mm was used, while the source to sample distance was 2.2 m. Changes in the shaped beamlets, with respect to a reference dataset without the sample in place, allow for the retrieval of sample properties: namely, transmission, refraction, and dark‐field or scattering.^15^ The transmission and refraction contrast channels allow quantification of the imaginary and real part of the complex refractive index (n(x;λ)=1−δ(x;λ)+iβ(x;λ)), respectively, where λ is the x‐ray wavelength. Provided that the shaped beamlets are adequately sampled (i.e., a pixel size smaller than the beamlet projected width at the detector position is used) and that cross‐talk between beamlets can be kept below ≈ 20% (i.e., the projected focal spot size is smaller than the mask period), the system resolution is exclusively determined by the mask aperture width, rather than source size and detector pixel pitch as it is the case for other x‐ray imaging modalities. This resolution limit of imaging systems based on intensity‐modulation masks has been theoretically^17^ and experimentally demonstrated.^18^ A recent experiment, performed with the microscope described in this note and with a mask with smaller aperture width (1.5 µm), showed the increase in resolution expected from the aperture width reduction.^19^ It is important to note that the resolution limit of the microscope discussed so far is only applicable to the direction of phase sensitivity, that is, orthogonally to the mask apertures. In the direction parallel to the mask slits, resolution is limited by the projected source size and detector pixel pitch. In fact, the one‐dimensional nature of the structured illumination of this set‐up creates a preferential direction both in terms of phase sensitivity and spatial resolution. This is particularly suited for imaging structures which have a strong directionality, for example, the nervous system and collagen. Isotropic resolution and phase sensitivity could be achieved through sample (or mask) rotation around the optical axis, or by adapting a two‐dimensional structured illumination like that shown in refs. [20, 21] to a microscopy system, which would however lead to longer exposure times due to the reduced mask open fraction. Transmission and refraction are associated with detection of individual sample features above the system resolution limit, that is, the mask aperture width. Dark‐field,^22^, ^23^ on the other hand, is sensitive to ensembles of sample features below the system resolution: while features are not individually resolved, the presence of the ensemble is highlighted.

In order to achieve the aperture‐driven resolution demonstrated in ref. [18], it is necessary to translate the sample by a distance equal to the mask period in the direction perpendicular to the mask slits, acquire images at each step, and recombine them (a process we refer to as *dithering* ^24^). The size of the dithering step is bound to be equal to or greater than the Nyquist frequency associated with the aperture width, to avoid aliasing. The sample was dithered in 20 steps of 0.95 µm, for an overall number of 20 planar images with an exposure time set to 10 s. For the retrieval of the three contrast channels, amplitude, mean and variance of each beamlet were extracted with $\left(A_{s},\;\mu_{s},\;\sigma_{s}^{2}\right)$ and without $\left(A_{r},\;\mu_{r},\;\sigma_{r}^{2}\right)$ the sample in place. Transmission (*T*), refraction (*R*) and dark‐field (DF) were obtained from the measured beamlet parameters using the following relationships:

(1) $$\begin{array}{cc} T & =\frac{A_{s}}{A_{r}}=exp\;\left[-\frac{4\pi }{\lambda }\int_{O}\beta \left(x,z;\lambda \right)\;dz\;\right];\\ R & =\frac{\mu_{s}-\mu_{r}}{\Delta z}\approx \frac{\lambda }{2\pi }\frac{\partial }{\partial_{x}}\Phi =\frac{\lambda }{2\pi }\frac{\partial }{\partial_{x}}\int_{O}\delta \left(x,z;\lambda \right)\;dz;\\ DF & =\frac{\sigma_{s}^{2}-\sigma_{r}^{2}}{\Delta z^{2}}. \end{array}$$

where the explicit dependence of *T* and *R* on β and δ is shown. Φ is the phase shift imparted to beam by the sample and the integration has to be extended over the depth of the object along the optical axis.

### 2.4 Histology

Due to limitations in performing standard histology processing for this sample arising from the dehydration occurring in ethanol, the sample was stained as a whole with Alcian blue and nuclear fast red dyes and imaged with an optical microscope (Leica DMi1, Leica Microsystems). Specifically, the sample was briefly hydrated in deionized water and then stained in 1% Alcian blue (pH 1.0) for 15 min, rinsed briefly in deionizsed water and then counterstained with nuclear red stain for 2 min. The samples were rinsed in water before microscopy imaging.

## 3 RESULTS AND DISCUSSION

**FIGURE 2:**
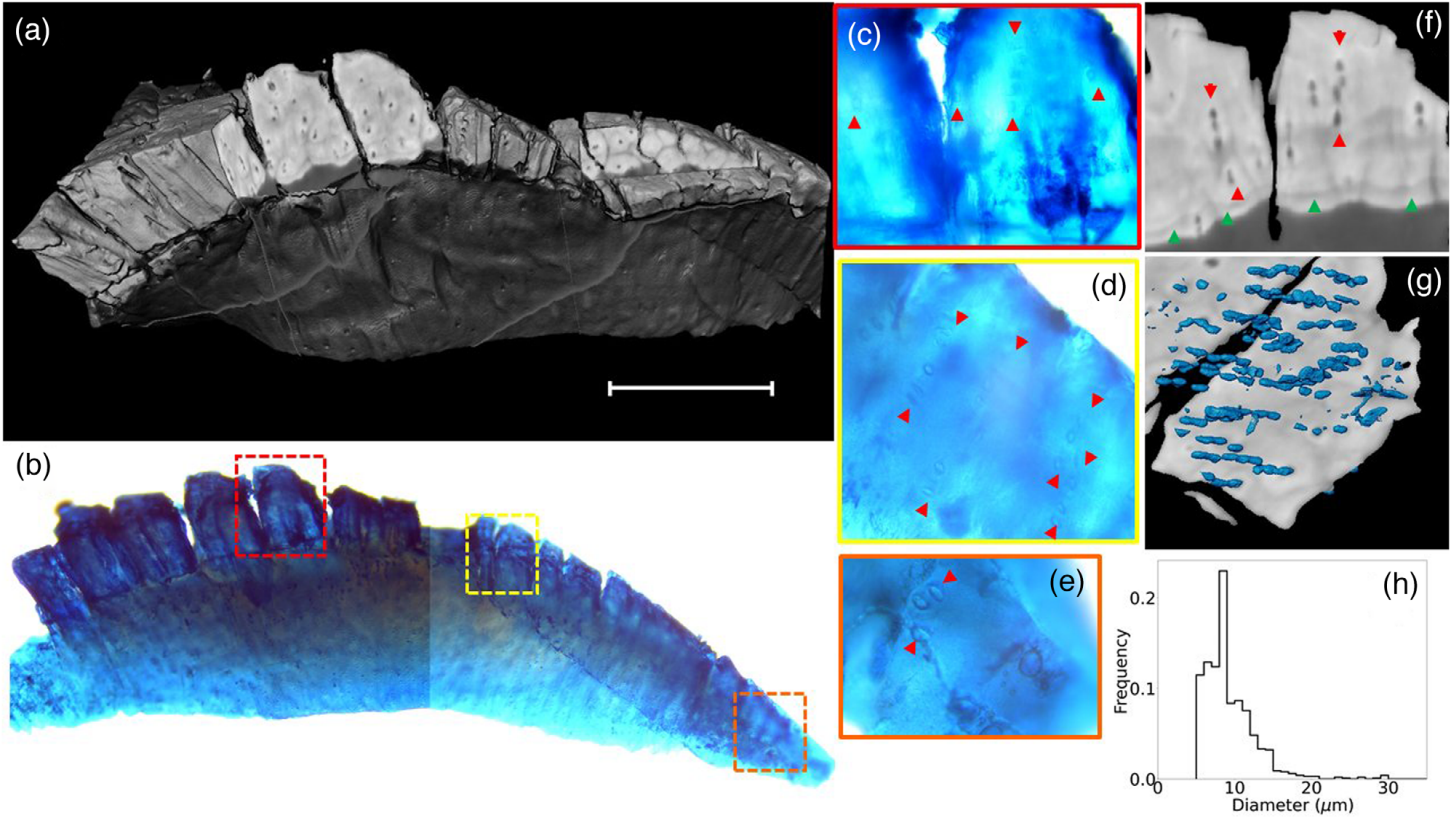
(a) Volumetric rendering of the cartilage sample from the DLS tomogram (scale bar 400 µm). Cut‐outs were extracted from the volume to allow the visualization of specific areas matching the histology. (b) Whole sample histological image, obtained by stitching two microscopy images due to a limited field of view. Specific areas, highlighted in the dashed boxes, are magnified in panels (c–e). Red arrowheads mark the columnar arrangement of chondrocytes. (f) Sagittal slice of the tomogram. Red arrowheads mark the columnar arrangement of chondrocytes. Green arrowheads identify the tidemark line. (g) Three‐dimensional visualization of segmented chondrocytes nuclei. (h) Distribution of nuclei diameter in the segmented chondrocytes.

A three‐dimensional representation of the tomogram acquired at DLS is shown in Figure 2a and compared with a similar view of the whole sample with histology stain (panel *b*). Magnified views of three regions of interest of the histology are reported in panels *c–e*. The uptake of the Alcian blue dye in the histology confirms the nature of the sample's tissue. Additionally, circular features visible in the histology can be identified as chondrocytes, due to their typical columnar alignment (marked with red arrowheads in the histology images), typical of deep cartilage layers. An analogous columnar arrangement for chondrocytes is highlighted in a reconstructed CT slice shown in panel *f*, matching the histology image of panel *c*. A feature that appeared to be the tidemark was visible (highlighted by green arrowheads in panel *f*) in the slice, identifying the transition between calcified and non‐calcified regions of the sample corresponding to the brighter (higher density) and darker (lower density) areas of the tomographic data, respectively. These findings are consistent with previously published literature,^25^ showing how the columnar arrangement of the chondrocytes is prominent just above the tidemark.

Chondrocyte nuclei were segmented in the calcified area of the sample using threshold‐based and watershed segmentation algorithms in Avizo (Thermo Fisher Scientific, version 2022.1). A three‐dimensional rendering of the segmented nuclei is shown in Figure 2g), highlighting the expected columnar architecture. The distribution of the measured diameter of nuclei was measured (see Figure 2h), resulting in a mean nucleus size of 10.8 ± 0.1 µm, calculated over 1145 segmented cells, comparable with results reported in literature.^26^, ^27^

**FIGURE 3:**
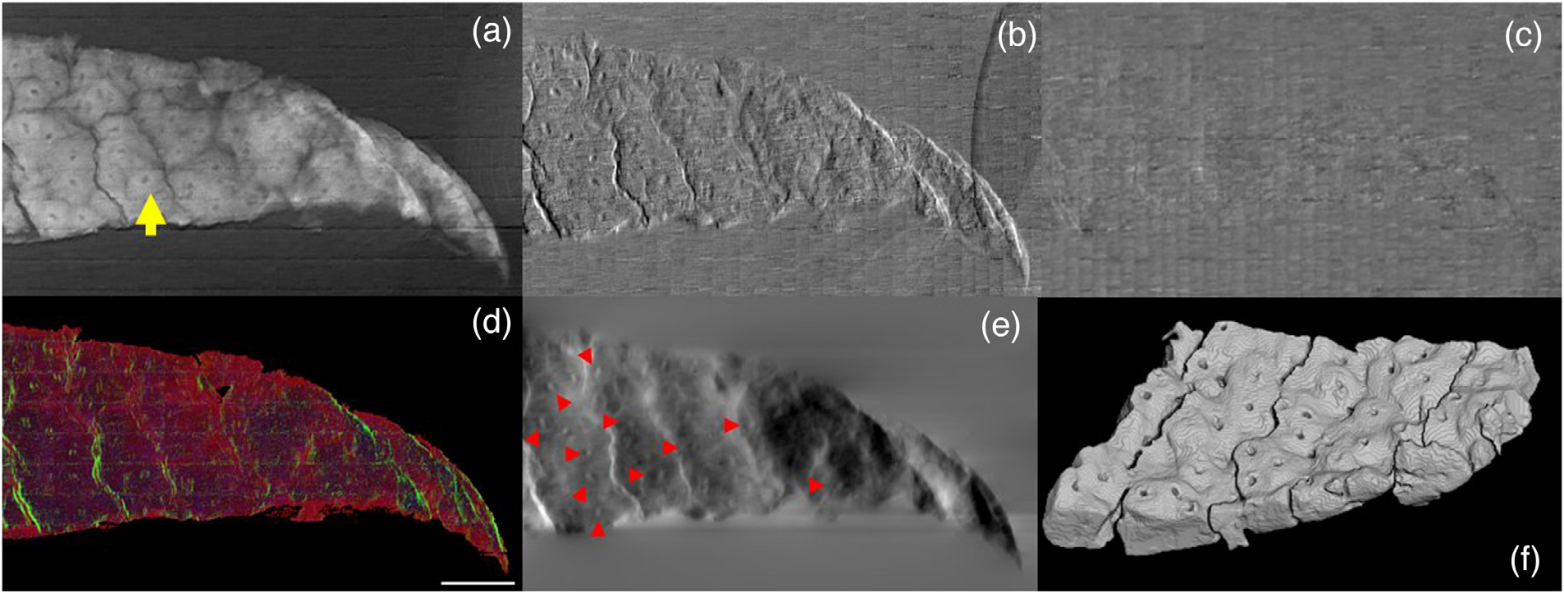
(a) Transmission, (b) refraction and (c) dark‐field retrieved images of the cartilage sample (scale bar 100 µm). (d) RGB image obtained by fusing images in panels (a–c) after intensity‐based segmentation. Red represents transmission, green the absolute value of refraction and blue scattering. (e) Integrated phase image. Red arrowheads mark chondrocytes' nuclei. (f) Volumetric rendering of the same area of the sample from the DLS tomogram. The yellow arrow in panel (a) identifies the chondrocyte shown in Figure 4.

Figure 3 shows the planar images obtained using the laboratory microscope, for the transmission (−log(T)), refraction and dark‐field contrast channels in panels *a‐c*. The transmission image reproduces the morphology of chondrocytes, including nucleus, cytoplasm and extra‐cellular matrix, in agreement with the DLS tomogram (Figure 3f). The refraction image highlights the edges of the cells' nuclei along the direction of sensitivity, that is, the one orthogonal to the mask apertures. The transmission image shows a higher contrast‐to‐noise ratio compared to refraction and dark After intensity‐based segmentation, the three contrast channels were fused to provide a single RGB image, with the three channels associated to transmission, absolute value of refraction and absolute value of dark‐field, respectively (panel *d*). The color rendering shows the complementarity of the three contrast channels. The refraction image was integrated^28^ to obtain the phase image (see Equation 1), shown in panel *e*, giving a clearer picture of the capability of the system to resolve chondrocytes' nuclei (highlighted by red arrowheads).

**FIGURE 4:**
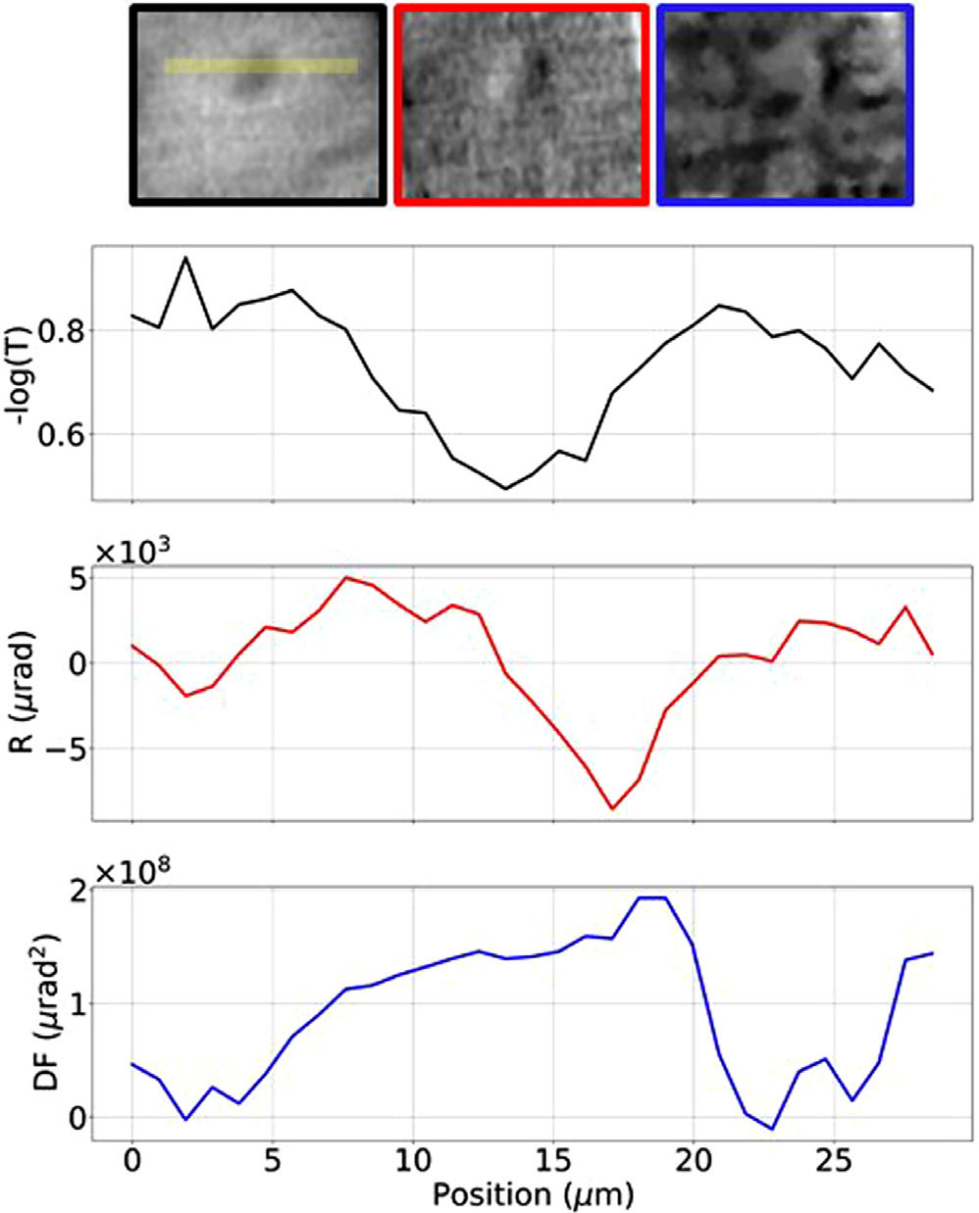
Detail of transmission (black), refraction (red) and dark‐field (blue) images for an individual chondrocyte. Intensity profiles for the channels are also shown. The dark‐field image is displayed at a different contrast level compared to Figure 3c to allow visualization of the cellular details.

Although sub‐cellular details are visible in all retrieved images, the transmission image (panel *a*) shows an increased contrast‐to‐noise ratio (CNR) compared to refraction and dark‐field (panels *b–c*), due to a higher background noise for the two phase‐based contrast channels arising from environmental instabilities over the timescale of the experiment. Some artifacts are visible in all retrieved images (*a–c*). Specifically, horizontal structured noise, oriented in the direction orthogonal to the mask slits, is visible in the retrieved images. This is due to the presence of absorbing bridges (or cross‐links) in the free‐standing mask supporting the open slits. Additionally, a brick‐like periodic noise is visible, which arises from the recombination of dithering steps in the presence of environmental vibrations that create a degree of inconsistency between successive steps. A magnified view of an individual chondrocyte (identified by a yellow arrow in Figure 3a) is provided in Figure 4, with relevant intensity profiles across the cell's nucleus. The transmission profile shows a variation in intensity, due to both variation in density between nucleus and cytoplasm and to the varying thickness of the cell. The differential refraction signal peaks at the nucleus' edges, highlighting a diameter of ≈ 10 µm, in agreement with the synchrotron‐based estimation. Finally, the dark‐field signal shows a positive value in the nuclear region, due to the presence of features below the resolution limit of the microscope.

## 4 CONCLUSIONS

This study provides the first proof‐of‐concept of imaging sub‐cellular features in cartilage with a laboratory‐based x‐ray microscope, validated with synchrotron data and gold standard histology. The presence of sub‐cellular structures in chondrocytes, such as nucleus and cytoplasm, can be revealed by making use of the complementarity of the retrieved contrast channels. It is important to note that, in this proof‐of‐concept work, only planar images were acquired with the laboratory‐based microscope. The suitability of the BT approach for tomographic reconstruction has been demonstrated before,^29^ and we expect the planned additional developments of the microscope to support tomographic imaging. The main limitation in the current system preventing tomographic imaging is the number of dithering steps to be acquired at each rotation angle, with this number being proportional to the mask period. Free‐standing masks with a shorter period (7.5 µm to be compared with 20 µm for the mask used in this work) were recently fabricated^19^ for the prototype microscope, drastically reducing the number of required dithering steps. Additionally, the use of alternative x‐ray optics, producing a smaller source focal spot, would allow reduction of the source to sample distance, leading to a higher flux and, thus, a reduction in total scan time. We expect the planned upgrades, combined with a cycloidal acquisition scheme,^30^ to allow for tomographic scans to be acquired in a relatively short scan time (≈ 1 h). The newly fabricated mask features bridges (responsible for the structured noise in Figure 3) placed every 400 µm, rather than 50 µm as in the mask used in this work, will largely reduce the structured noise in the retrieved images. Further developments could include the capability to image thicker samples through the use of higher energy monochromatic beams, for example, Ga Kα (9.2 keV) or Mo Kα (17.5 keV), which would lead to a gain in accessible tissue thickness of a factors 2 and 10, respectively.

## CONFLICT OF INTEREST STATEMENT

Dr J. Ferrara is a Rigaku employee; Rigaku has a potential interest in the commercial exploitation of the results presented in this abstract. Prof M. Endrizzi and Alessandro Olivo are named inventors on patents protecting the described technology. All other authors have no financial interests to disclose.

## Data Availability

The data that support the findings of this study are available from the corresponding author upon reasonable request.
